# Comparison of the 24-Style Tai Chi intervention based on various promotion approaches on college students’ mental health: A randomized controlled trial

**DOI:** 10.1371/journal.pone.0343808

**Published:** 2026-02-27

**Authors:** Kai Xu, Zetong Sun, Defeng Dong, Zhiyuan Tan, Jundi Chen, Carlo Castagna, Peter Krustrup, Jun Xu, Shuang Wei

**Affiliations:** 1 School of Sport and Art, Shandong Sport University, Jinan, China; 2 The School of Human Science, The University of Western Australia, Perth, Australia; 3 School of Graduate Education, Shandong Sport University, Jinan, China; 4 Faculty of Sport and Physical Education, University of Belgrade, Belgrade, Serbia; 5 Guangdong Lingnan Institute of Technology, Guangzhou, China; 6 Department of Education and Sport Sciences, Pegaso Telematic University, Naples, Italy; 7 Football Training and Biomechanics Laboratory, Technical Department FIGC, Coverciano, Italy; 8 Department of Sports Science and Clinical Biomechanics, SDU Sport and Health Sciences Cluster (SHSC), University of Southern Denmark, Odense, Denmark; 9 Danish Institute for Advanced Study (DIAS), University of Southern Denmark, Odense, Denmark,; 10 Sport and Health Sciences, University of Exeter, Exeter, United Kingdom; University of Rwanda College of Medicine and Health Sciences, RWANDA

## Abstract

**Background:**

The escalating pace of academic pressures and social life have intensified mental health challenges among college students, including widespread anxiety and depression. Non-pharmacological interventions, such as exercise therapy, have gained prominence, with 24-Style Tai Chi emerging as a promising mind-body exercise due to its simplicity and potential mental health benefits. However, its effectiveness across various promotion approaches in higher education settings remains underexplored.

**Objective:**

This study aimed to evaluate the impact of different 24-Style Tai Chi promotion methods (on-site, online, mixed, and independent practice) on college students’ mental health, assess intervention efficacy, and identify an optimal promotion strategy.

**Methods:**

A randomized controlled trial (RCT) involving 250 college students from Shandong Sport University was conducted over 8 weeks. Participants were allocated to On-Site Promotion Group (Offline-PG), Online Promotion Group (Offline-PG), Mixed Promotion Group (MPG), Independent Practice Group (IPG), or Control Group (CG). Mental health was assessed using the Self-Rating Anxiety Scale (SAS), Self-Rating Depression Scale (SDS), and General Self-Efficacy Scale (GSES) at baseline, 4 and 8 weeks, and follow-up. Data were analyzed using one-way ANOVA, repeated measures ANOVA (RM-ANOVA), and regression analysis to evaluate intervention effects and participation impacts.

**Results:**

Offline-PG (90% attendance) and MPG (84% attendance) showed significant reductions in anxiety (SAS: Offline-PG 34.8 ± 6.3, MPG 36.2 ± 7.0, *P* < 0.01) and depression (SDS: Offline-PG 33.7 ± 6.9, MPG 34.9 ± 7.2, *P* < 0.05) by week 8. Online-PG (72% attendance) and MPG demonstrated significant self-efficacy improvements (GSES: Online-PG 36.1 ± 5.2, MPG 35.5 ± 5.6, *P* < 0.01). IPG (60% attendance) showed no significant changes. ANOVA revealed inter-group differences (SAS F = 6.45, *P* = 0.004; SDS F = 5.32, *P* = 0.009; GSES F = 6.74, *P* = 0.003), with RM-ANOVA confirming time effects. Regression analysis indicated participation strongly correlated with anxiety and depression reductions in Offline-PG (R = 0.62, *P* = 0.003) and MPG (R = 0.58, *P* = 0.004) and self-efficacy gains in Online-PG and MPG (R = 0.67, *P* = 0.002).

**Conclusion:**

24-Style Tai Chi effectively improves college students’ mental health, with on-site and mixed approaches excelling in reducing anxiety and depression, and online and mixed approaches enhancing self-efficacy. A hybrid promotion strategy is recommended to optimize participation and outcomes.

**Registration number:** TCTR20250306005; https://thaiclinicaltrials.org/.

## Introduction

In the context of the enhancing academic pressure and the accelerating pace of modern social life, mental health problems have become more prominent for college students. Emotional disorders, such as anxiety, and depression significantly impact their academic performance, social abilities, and future career development. Existing literature has reported that although medication, and psychotherapy can positively delay certain symptoms, problems linked to side impacts, and patient compliance persist [1,2]. Research has shown that the side effects and adherence issues of pharmacological treatments require further investigation [2–4]. Hence, non-pharmacological interventions have obtained increasing attention, particularly mind-body exercises, such as Tai Chi. Literature has displayed that Tai Chi, an conventional mind-body intervention approach, has significant positive impacts on enhancing mental health, particularly in relieving symptoms of anxiety, and depression [3,5–7]. These articles stress that exercise interventions, such as Tai Chi, not only enhance negative emotions, but also have the possible role to enhance college students’ psychological resilience, acting as an effective supplement in mental health interventions [4]. in the context of integrating physical movement, breathing regulation, and mental focus, Tai Chi causes overall mental health enhancement, making it a safe, and popular non-pharmacological intervention.

As a simplified form of Tai Chi, 24-Style Tai Chi is particularly suitable for college students due to its ease of learning, simplicity of practice, and low time requirements. It has been reported in the literature that for college students, adolescents, and healthy people under stress, 24-style Taijiquan can effectively delay negative emotions such as anxiety and depression, and enhance psychological resilience and self-efficacy [8–10]. A systematic review concluded that Tai Chi has significant positive impacts on college students’ anxiety, depression, and overall mental health [9,11,12]. Besides, Tai Chi has been reported to help college students cope with psychological stress in the context of enhancing cognitive functions of the brain, particularly in executive control, and attention [9]. However, although the verified mental health benefits, its promotion, and application in colleges and universities remain limited, revealing a need for more research to investigate its effectiveness in diverse educational settings.

Therefore, our study aims to investigate the promotion models of 24-Style Tai Chi in the context of college students, analyze the intervention impacts of various promotion approaches on students’ mental health, and further propose strengthened promotion strategies. Our study aims to investigate the feasibility of implementing 24-Style Tai Chi in colleges via various promotion approaches, and its intervention impacts on college students’ mental health. Specific objectives include: (1) analyzing the impact, and sustainability of various promotion approaches (online, On-Site, mixed models) on college students’ participation in Tai Chi; (2) proposing a strengthened strategy for the long-term promotion of Tai Chi in colleges, and universities.

## Methods

### Study design

This study adopted a randomized controlled trial (RCT) design, with college students from Shandong Sport University as participants. Recruitment and follow-up were conducted between March 4, 2025 and May 16, 2025. The study strictly adhered to the ethical principles of the World Medical Association Declaration of Helsinki. The protocol was reviewed and approved by the Biomedical Ethics Committee of Shandong Sport University (Approval No.: 2025027). All participants provided written informed consent before participation.

The trial was prospectively registered in the Thai Clinical Trial Registry (TCTR) under the registration number TCTR20250306005 (https://www.thaiclinicaltrials.org/show/TCTR20250306005; registered on March 6, 2025), prior to the commencement of participant recruitment.

The authors confirm that all ongoing and related trials for this intervention are registered.

### Sample selection

The sample size was determined a priori using GPower software (version 3.1.9.7) [1]. Based on previous RCTs examining the effect of Tai Chi on anxiety and depression in student populations [2,3], we anticipated a medium-to-large effect size (Cohen’s f = 0.30). For a one-way ANOVA with five groups, a significance level (α) of 0.05, and a statistical power (1-β) of 0.80, the analysis indicated a minimum total sample size of 200. To account for a potential attrition rate of approximately 20%—estimated from similar longitudinal exercise intervention studies [4]—we aimed to recruit 250 participants (50 per group), ensuring sufficient power for the primary analyses even after dropouts. To enhance the representativeness of the study, and control experimental errors, it was planned to recruit 50 college students per group, causing a total sample size of 250. This estimation determines sufficient statistical power for multi-group comparisons, and analyses of various promotion approaches. Hence, 250 college students were randomly recruited from the university as research subjects, with 50 participants per group. All participants signed informed consent forms before the experiment.

### Grouping protocol

**Fig 1:**
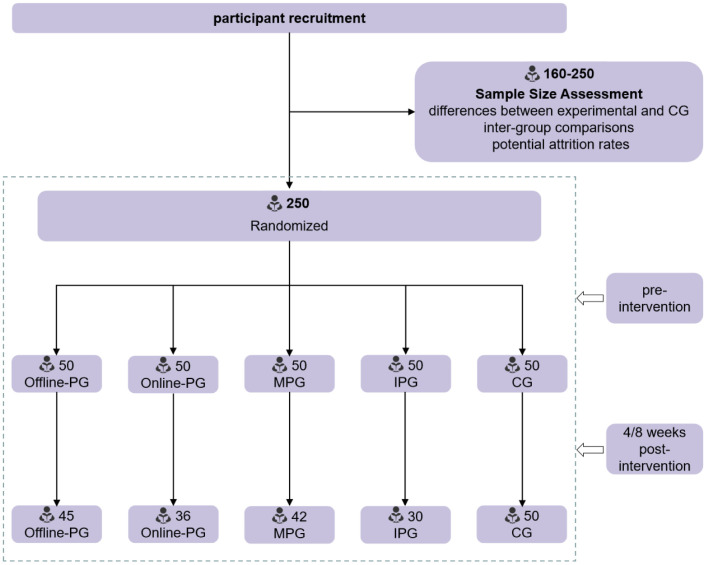
Recruitment Experiment Design Flowchart, Flowchart illustrating the research selection and screening process.

(1) Offline-PG: Organized via university physical education courses or Tai Chi clubs, with 3 on-site Tai Chi sessions per week, each lasting 60 minutes, guided in the context of professional Tai Chi instructors; (2) Online-PG: Participants evaluated 24-Style Tai Chi instructional videos via an online platform, and practiced independently at least 3 times per week, 60 minutes per session. The system recorded practice duration, and frequency; (3) MPG: A combination of online, and On-Site approaches, including 1 On-Site session, and 2 online independent practice sessions per week, with the same schedule as other groups; (4) IPG: Participants were provided with 24-Style Tai Chi practice guidelines, and instructional materials, and practiced independently in the context of their own schedules without supervision; (5) CG: No Tai Chi practice was conducted, and participants sustained their daily activities.The detailed process is illustrated in Fig 1.

### Data collection, and measurement tools

(1) Mental health analysis: Standardized scales were utilized to analyze participants’ mental health before the intervention, at 4 weeks, 8 weeks after the intervention, and during follow-up, including the SAS [13], SDS [14] and GSES [15]. (2) Participation analysis: The participation, and sustainability of various promotion approaches were assessed via records from the online platform, and attendance rates of On-Site courses

### Blinding implementation

To decrease potential bias, and determine the objectivity, and accuracy of the study results, a single-blind method was employed. Specifically, all participants were unaware of their assigned intervention group when allocated to various promotion approaches, i.e., they did not determine whether they were randomized to the online or On-Site groups, MPG, or IPG. They only received the study arrangement, and participated in the corresponding 24-Style Tai Chi practice or sustained daily life (CG). Participants were only informed that the study was linked to mental health, and exercise intervention, and researchers would conduct regular mental health analysis. Moreover, testers responsible for mental health analysis, and data analysts were also blinded, as they were unaware of which intervention group each participant belonged to, determing that their analysis, and data analysis were not impact in the context of group information. This single-blind design lowers observer impacts, and participants’ psychological expectation impacts, determining the fairness, and validity of the study results.

### Data analysis

All data are reported as “mean ± standard deviation (Mean ± SD)”. Multiple statistical approaches were utilized to test differences in the impacts of 24-Style Tai Chi on college students’ mental health (anxiety, depression, self-efficacy, etc.) under various promotion approaches. Descriptive statistics were utilized to outline the basic demographic features, and pre-intervention mental health scores of each group to determine inter-group balance. Besides, one-way analysis of variance (ANOVA) [16] was utilized to compare overall differences in mental health indicators (anxiety, depression, self-efficacy) between various promotion groups, and CG after the intervention. If significant differences were found, post-hoc tests were conducted to identify specific inter-group differences. Besides, analysis of covariance (ANCOVA) [17] was utilized to adjust for the impact of potential covariates (e.g., gender, age, baseline mental health levels) to improve the accuracy of results. For time impact analysis of each group (pre-intervention, mid-intervention, post-intervention), RM-ANOVA [18] was employed to analyze the altering trends of mental health indicators under various promotion approaches. Finally, regression analysis was utilized to investigate the association between participation in promotion approaches, and mental health enhancement, further revealing the potential mechanisms of intervention impacts. All analyses were performed utilizing SPSS 26 statistical software [19], with a significance level set at *P* < 0.05 (two-tailed test). The significance level was set at p < 0.05 for all two-tailed tests. To control the increased risk of Type I errors due to multiple comparisons in the post-hoc analyses (following a significant ANOVA result) and in the correlation analyses, the Bonferroni correction was applied. This method adjusts the significance level by dividing it by the number of comparisons being made. Specific adjusted alpha levels are detailed in the footnotes of the respective results tables.

The primary aim of the statistical analysis was to evaluate the differential effectiveness of the various promotion approaches. Therefore, the analytical strategy was designed to make comparisons both between the active intervention groups and against the control group. First, a one-way ANOVA was conducted to assess the overall differences across all five groups (the four intervention groups and the control group) at the primary endpoint (Week 8). If the ANOVA revealed a significant overall effect, post-hoc tests (e.g., Dunnett’s test or Bonferroni correction) were planned to make specific comparisons. The use of Dunnett’s test was specified a priori for comparisons of each intervention group against the single control group, while other post-hoc tests were used for interventional group comparisons. Furthermore, Analysis of Covariance (ANCOVA) was also planned to adjust for baseline scores and other potential covariates to improve the precision of the treatment effect estimates

## Results

### Participation analysis, and basic sample information

The participation and sustainability of each promotion method were evaluated in the context of recording online practice duration, and On-Site course attendance, with the following specific results: (1) The average attendance rate of Offline-PG was 90%, higher than that of other groups. Most participants sustained at least twice-weekly participation during follow-up. (2) The participation frequency of Online-PG reduced over time, with the average participation rate dropping to 72% after 8 weeks. (3) The participation rate of MPG was 84%, sustaining a high fraction of participants during follow-up. (4) The participation rate of IPG was relatively low, at roughly 60%, with several participants discontinuing practice midway. via descriptive statistical analysis, there were no significant differences in demographic features, such as gender, age, and baseline mental health scores in the context of groups (Offline-PG, Online-PG, MPG, IPG, and CG) before the intervention. The mean baseline SAS score was 44.3 ± 8.2, the mean SDS score was 46.1 ± 9.5, and the mean GSES score was 29.7 ± 5.8. Inter-group t-test results reported no significant differences in SAS (*P* = 0.72), SDS (*P* = 0.68), or GSES (*P* = 0.74) scores in the context of groups at baseline. A comparison of 24-Style Tai Chi participation, and baseline mental health features under various promotion approaches is reported in Table 1.

**Table 1:** Comparison of 24-Style Tai Chi Participation, and Baseline Mental Health features Under various Promotion approaches.

Group	Offline-PG	Online-PG	MPG	IPG	CG
Average Attendance/Participation Rate	90%	72%	84%	60%	N/A
Original/Final Number of Participants	50/45	50/36	50/42	50/30	50/50
Participation in Follow-up Phase	Most participants maintained participation at least twice a week	Participation frequency decreased over time	Maintained a high level of participation	Some participants dropped out midway	No intervention
SAS Baseline Score (M ± SD)	44.1 ± 8.0	44.5 ± 8.3	44.4 ± 8.1	44.2 ± 8.5	44.3 ± 8.4
SDS Baseline Score (M ± SD)	46.3 ± 9.3	46.0 ± 9.6	46.4 ± 9.4	46.1 ± 9.7	46.2 ± 9.5
GSES Baseline Score (M ± SD)	29.6 ± 5.7	29.8 ± 5.9	29.9 ± 5.6	29.5 ± 5.8	29.7 ± 5.8
Inter-group t-test P-value (SAS)	> 0.05	> 0.05	> 0.05	> 0.05	> 0.05
Inter-group t-test P-value (SDS)	> 0.05	> 0.05	> 0.05	> 0.05	> 0.05
Inter-group t-test P-value (GSES)	> 0.05	> 0.05	> 0.05	> 0.05	> 0.05

### Changes in mental health indicators

At 4 weeks, 8 weeks after the intervention, and during the follow-up period, all experimental groups (Offline-PG, Online-PG, MPG, IPG) reported varying degrees of enhancement in mental health. Specific result data are reported in Table 2.

**Table 2:** Impact of 24-Style Tai Chi on Changes in College Students’ Mental Health Indicators Under various Promotion approaches.

Mental Health Indicators	Offline-PG(n = 45)	Online-PG(n = 36)	MPG(n = 42)	IPG(n = 30)	CG(n = 50)
SAS Score (4 weeks)	38.9 ± 6.8	41.3 ± 7.6	39.7 ± 7.2	41.7 ± 7.9	44.3 ± 8.4
SAS Score (8 weeks)	34.8 ± 6.3	39.5 ± 7.8	36.2 ± 7.0	40.1 ± 8.0	44.3 ± 8.4
SAS Score (Follow-up)	35.1 ± 6.5	40.2 ± 7.9	36.5 ± 7.1	40.3 ± 8.2	44.3 ± 8.4
SDS Score (4 weeks)	37.4 ± 7.2	40.5 ± 7.8	38.2 ± 7.5	40.9 ± 7.9	46.2 ± 9.5
SDS Score (8 weeks)	33.7 ± 6.9	38.6 ± 7.5	34.9 ± 7.2	39.3 ± 7.9	46.2 ± 9.5
SDS Score (Follow-up)	34.0 ± 7.1	39.0 ± 7.6	35.1 ± 7.3	39.4 ± 8.0	46.2 ± 9.5
GSES Score (4 weeks)	31.7 ± 5.8	33.9 ± 5.5	33.7 ± 5.4	30.8 ± 5.8	29.7 ± 5.8
GSES Score (8 weeks)	32.8 ± 5.9	36.1 ± 5.2	35.5 ± 5.6	31.5 ± 6.1	29.7 ± 5.8
GSES Score (Follow-up)	32.7 ± 5.8	35.8 ± 5.3	35.3 ± 5.5	31.6 ± 6.0	29.7 ± 5.8

### Changes in anxiety levels (SAS scores)

On the SAS, anxiety scores in each experimental group commonly reduced with the intervention, but the magnitude of enhancement varied significantly: (1) Offline-PG: SAS scores reduced to 38.9 ± 6.8 at week 4, reporting a significant reduction compared to the baseline (*P* = 0.015 < 0.05), and further dropped to 34.8 ± 6.3 at week 8 (*P* = 0.004), determining significant enhancement. SAS scores remained stable at 35.1 ± 6.5 during the follow-up period. (2) Online-PG: SAS scores were 41.3 ± 7.6 at week 4, with a slight, but non-significant enhancement compared to the baseline (*P* = 0.095), and 39.5 ± 7.8 at week 8 (*P* = 0.12), being unable to reach a significant level. Scores slightly rebounded to 40.2 ± 7.9 during follow-up. (3) MPG: SAS scores reduced to 39.7 ± 7.2 at week 4, significantly lower than the baseline (*P* = 0.023 < 0.05), and 36.2 ± 7.0 at week 8 (*P* = 0.007 < 0.01), also reporting significant enhancement in anxiety. Scores stabilized at 36.5 ± 7.1 during follow-up. (4) IPG: SAS scores were 41.7 ± 7.9 at week 4, with no significant change compared to the baseline (*P* = 0.18 > 0.05), and 40.1 ± 8.0 at week 8 (*P* = 0.15 > 0.05), with no significant enhancement. Scores remained at 40.3 ± 8.2 during follow-up.

### Changes in depression levels (SDS scores)

On the SDS, depression levels in each experimental group also improved, particularly in Offline-PG, and MPG: (1) Offline-PG: SDS scores were 37.4 ± 7.2 at week 4, significantly lower than the baseline (*P* = 0.021 < 0.05), and dropped to 33.7 ± 6.9 at week 8 (*P* = 0.009 < 0.01), determining significant enhancement in depression. Scores slightly rebounded to 34.0 ± 7.1 during follow-up. (2) Online-PG: SDS scores reduced to 40.5 ± 7.8 at week 4, with no significant change compared to the baseline (*P* = 0.102 > 0.05), and 38.6 ± 7.5 at week 8 (*P* = 0.11 > 0.05), with no significant enhancement in depression. Scores slightly rebounded to 39.0 ± 7.6 during follow-up. (3) MPG: SDS scores were 38.2 ± 7.5 at week 4, significantly lower than the baseline (*P* = 0.028 < 0.05), and 34.9 ± 7.2 at week 8 (*P* = 0.013 < 0.05), determining significant enhancement in depressive symptoms. Scores remained stable at 35.1 ± 7.3 during follow-up. (4) IPG: SDS scores were 40.9 ± 7.9 at week 4, with no significant change compared to the baseline (*P* = 0.16 > 0.05), and 39.3 ± 7.9 at week 8 (*P* = 0.16 > 0.05), with no significant enhancement in depression. Scores stabilized at 39.4 ± 8.0 during follow-up.

### Changes in self-efficacy (GSES Scores)

Changes in GSES scores determined that Online-PG, and MPG had significant impacts in enhancing self-efficacy: (1) Offline-PG: GSES scores were 31.7 ± 5.8 at week 4, with no significant difference compared to the baseline (*P* = 0.08 > 0.05), and 32.8 ± 5.9 at week 8 (*P* = 0.09 > 0.05), with a small enhancement that fail to obtain a significant level. Scores remained stable during follow-up. (2) Online-PG: GSES scores enhanced to 33.9 ± 5.5 at week 4, significantly higher than the baseline (*P* = 0.012 < 0.05), and reached 36.1 ± 5.2 at week 8 (*P* = 0.003 < 0.01), determining extremely significant enhancement in self-efficacy. Scores remained at 35.8 ± 5.3 during follow-up. (3) MPG: GSES scores were 33.7 ± 5.4 at week 4, significantly higher than the baseline (*P* = 0.018 < 0.05), and enhanced to 35.5 ± 5.6 at week 8 (*P* = 0.005 < 0.01), reporting significant enhancement in self-efficacy. Scores remained stable at 35.3 ± 5.5 during follow-up. (4) IPG: GSES scores were 30.8 ± 5.8 at week 4, with no significant enhancement (*P* = 0.14 > 0.05), and 31.5 ± 6.1 at week 8 (*P* = 0.13 > 0.05), with a small enhancement in self-efficacy that did not reach a significant level. Scores fluctuated slightly during follow-up.

### Inter-group difference analysis

One-way ANOVA was utilized to compare the overall differences in mental health indicators, such as anxiety (SAS), depression (SDS), and self-efficacy (GSES) in the context of groups, analyzing the differences in effectiveness of various promotion approaches in enhancing college students’ mental health. The analysis of differences in mental health indicators in the context of groups under various promotion approaches is reported in Table 3.

**Table 3:** Analysis of Differences in Mental Health Indicators in the context of Groups Under various Promotion approaches.

Comparison	Anxiety (SAS Score)	Depression (SDS Score)	Self-Efficacy (GSES Score)
One-Way ANOVA (Overall)	F = 6.45, p = .004	F = 5.32, p = .009	F = 6.74, p = .003
Post-hoc p-values vs. Control Group (CG)			
Offline-PG vs. CG	.004	.009	.090
Online-PG vs. CG	.120	.110	.003
MPG vs. CG	.007	.013	.005
IPG vs. CG	.150	.160	.130
Between Intervention Groups			
Offline-PG vs. Online-PG	.018	.025	.010
Offline-PG vs. MPG	.210	.185	.022
Offline-PG vs. IPG	.002	.001	.135
Online-PG vs. MPG	.045	.055	.285
Online-PG vs. IPG	.320	.275	.001
MPG vs. IPG	.009	.007	.005

Footnote for Table 3:

Abbreviations: PG, Promotion Group; MPG, Mixed Promotion Group; IPG, Independent Practice Group; CG, Control Group.

The one-way ANOVA was used to test the overall effect across all five groups. Post-hoc pairwise comparisons were conducted only if the overall ANOVA was significant. To account for multiple comparisons, a Bonferroni correction was applied, setting the family-wise significance level at p < 0.05. The specific number of comparisons and adjusted alpha levels were as follows: for the four pre-planned comparisons of each intervention group vs. the control group, the adjusted significance level was p < 0.0125 (0.05/4); for all possible pairwise comparisons between groups, the adjusted level was p < 0.005 (0.05/10). Unadjusted p-values are presented in the table, with asterisks denoting significance after Bonferroni correction: p < 0.0125, p < 0.005.

### Inter-group differences in anxiety levels (SAS Scores)

At week 8, there were significant differences in SAS scores in the context of groups with various promotion approaches (ANOVA result: F = 6.45, *P* = 0.004 < 0.01). Specific inter-group comparisons reported: (1) Offline-PG vs. CG: Anxiety scores reduced significantly, with a significant difference (*P* = 0.004 < 0.01). (2) Online-PG vs. CG: Anxiety scores reduced, but the difference was not significant (*P* = 0.12 > 0.05). (3) MPG vs. CG: Anxiety scores reduced significantly, with a significant difference (*P* = 0.007 < 0.01). (4) IPG vs. CG: The change in anxiety scores was not significant (*P* = 0.15 > 0.05).

### Inter-group differences in depression levels (SDS scores)

At week 8, SDS scores reported significant differences in the context of groups with various promotion approaches (ANOVA result: F = 5.32, *P* = 0.009 < 0.01). Specific inter-group comparison results were as follows: (1) Offline-PG vs. CG: Depression scores reduced significantly, with a significant difference (*P* = 0.009 < 0.01). (2) Online-PG vs. CG: Depression scores reduced slightly, but the difference was not significant (*P* = 0.11 > 0.05). (3) MPG vs. CG: Depression scores reduced significantly, with a significant difference (*P* = 0.013 < 0.05). (4) IPG vs. CG: There was no significant change in depression scores (*P* = 0.16 > 0.05).

### Inter-group differences in self-efficacy (GSES Scores)

At week 8, there were also significant differences in self-efficacy (GSES) in the context of groups (ANOVA result: F = 6.74, *P* = 0.003 < 0.01). Specific inter-group comparisons were as follows: (1) Offline-PG vs. CG: Self-efficacy enhanced slightly, but the difference was not significant (*P* = 0.09 > 0.05). (2) Online-PG vs. CG: Self-efficacy enhanced significantly, with a significant difference (*P* = 0.003 < 0.01). (3) MPG vs. CG: Self-efficacy enhanced significantly, with a significant difference (*P* = 0.005 < 0.01). (4) IPG vs. CG: There was no significant change in self-efficacy (*P* = 0.13 > 0.05).

### Time impact analysis

RM-ANOVA was utilized to analyze the altering trends of anxiety (SAS), depression (SDS), and self-efficacy (GSES) scores in each group before the intervention, at 4 weeks, 8 weeks, and during the follow-up period. The time impact analysis of mental health indicators in each group under various promotion approaches is reported in Table 4.

**Table 4:** Time impact Analysis of Mental Health Indicators in Each Group Under various Promotion approaches.

Group	Anxiety (SAS)	Depression (SDS)	Self-Efficacy (GSES)
Time Effect (RM-ANOVA)			
Offline-PG	F = 8.22, p = .003	F = 7.93, p = .002	F = 2.05, p = .090
Online-PG	F = 2.11, p = .090	F = 2.23, p = .080	F = 7.58, p = .004
MPG	F = 7.45, p = .004	F = 6.89, p = .005	F = 6.45, p = .006
IPG	F = 1.98, p = .110	F = 1.77, p = .120	F = 1.98, p = .100

Footnote for Table 4:

RM-ANOVA = Repeated Measures Analysis of Variance.

For the correlation analysis in Table 5, a Bonferroni correction was applied to control for the three primary mental health outcomes (anxiety, depression, self-efficacy) tested within each group. The adjusted significance level was set at p < 0.0167 (0.05/3). Unadjusted p-values are shown, with significance after correction denoted as p < 0.0167.

**Table 5:** Analysis of the impact Mechanism of Participation on Mental Health enhancement Under various Promotion approaches.

Group	Anxiety (SAS)	Depression (SDS)	Self-Efficacy (GSES)	Group
Offline-PG	R = 0.62, p = .003	R = 0.58, p = .004	R = 0.29, p = .080	Offline-PG
Online-PG	R = 0.32, p = .090	R = 0.30, p = .100	R = 0.67, p = .002	Online-PG
MPG	R = 0.59, p = .004	R = 0.55, p = .005	R = 0.65, p = .003	MPG

Footnote for Table 5:

RM-ANOVA = Repeated Measures Analysis of Variance.

### Time impact on anxiety levels (SAS Scores)

RM-ANOVA reported that Offline-PG, and MPG had significant time impacts on anxiety scores (SAS), while Online-PG, and IPG had no significant time impacts. Specific results were as follows: (1) Offline-PG: SAS scores commonly reduced over time, reaching 38.9 ± 6.8 at 4 weeks, further dropping to 34.8 ± 6.3 at 8 weeks, and sustaining at 35.1 ± 6.5 during the follow-up period. The time impact was significant (F = 8.22, *P* = 0.003 < 0.01), determing that anxiety levels enhanced significantly over time. (2) Online-PG: SAS scores reduced slightly in the first 4 weeks, but stabilized after 8 weeks, with scores of 41.3 ± 7.6 at 4 weeks, and 39.5 ± 7.8 at 8 weeks, and a slight rebound to 40.2 ± 7.9 during the follow-up period. The time impact was not significant (F = 2.11, *P* = 0.09 > 0.05), with no significant change in anxiety levels. (3) MPG: SAS scores reduced from a baseline of 44.4 ± 8.1 to 39.7 ± 7.2 at 4 weeks, and 36.2 ± 7.0 at 8 weeks, sustaining at 36.5 ± 7.1 in the context of the follow-up period. The time impact was significant (F = 7.45, *P* = 0.004 < 0.01), reporting continuous enhancement in anxiety levels. (4) IPG: SAS scores reported no significant change during the intervention period, with scores of 41.7 ± 7.9 at 4 weeks, and 40.1 ± 8.0 at 8 weeks, and 40.3 ± 8.2 during the follow-up period. The time impact was not significant (F = 1.98, *P* = 0.11 > 0.05).

### Time impact on depression levels (SDS scores)

In terms of depression scores (SDS), Offline-PG, and MPG reported significant time impacts, while Online-PG, and IPG had no significant time impacts on depression enhancement. Specific results were as follows: (1) Offline-PG: SDS scores reduced to 37.4 ± 7.2 at 4 weeks, further dropping to 33.7 ± 6.9 at 8 weeks, and slightly rebounding to 34.0 ± 7.1 during the follow-up period. The time impact was significant (F = 7.93, *P* = 0.002 < 0.01), determining significant enhancement in depression levels. (2) Online-PG: SDS scores were 40.5 ± 7.8 at 4 weeks, 38.6 ± 7.5 at 8 weeks, and slightly rebounded to 39.0 ± 7.6 during the follow-up period, with a small change range. The time impact was not significant (F = 2.23, *P* = 0.08 > 0.05). (3) MPG: SDS scores commonly reduced from a baseline of 46.4 ± 9.4 to 38.2 ± 7.5 at 4 weeks, and 34.9 ± 7.2 at 8 weeks, sustaining at 35.1 ± 7.3 during the follow-up period. The time impact was significant (F = 6.89, *P* = 0.005 < 0.01), determining that depression levels enhanced significantly over time. (4) IPG: SDS scores reported little change at 4 weeks, and 8 weeks, with scores of 40.9 ± 7.9, and 39.3 ± 7.9 respectively, and sustained at 39.4 ± 8.0 during the follow-up period. The time impact was not significant (F = 1.77, *P* = 0.12 > 0.05).

### Time impact on self-efficacy (GSES scores)

Time impact analysis of GSES scores reported that self-efficacy in Online-PG, and MPG enhanced significantly over time, while the time impacts in Offline-PG, and IPG were not significant. Specific results were as follows: (1) Offline-PG: GSES scores were 31.7 ± 5.8 at 4 weeks, and 32.8 ± 5.9 at 8 weeks, with a small change range (F = 2.05, *P* = 0.09 > 0.05), and the time impact was not significant. (2) Online-PG: GSES scores enhanced from a baseline of 29.8 ± 5.9 to 33.9 ± 5.5 at 4 weeks, reaching 36.1 ± 5.2 at 8 weeks, and sustaining at 35.8 ± 5.3 during the follow-up period. The time impact was significant (F = 7.58, *P* = 0.004 < 0.01), reporting significant enhancement in self-efficacy. (3) MPG: GSES scores enhanced from a baseline of 29.9 ± 5.6 to 33.7 ± 5.4 at 4 weeks, and 35.5 ± 5.6 at 8 weeks, sustaining at 35.3 ± 5.5 during the follow-up period. The time impact was significant (F = 6.45, *P* = 0.006 < 0.01), with self-efficacy enhancing significantly over time. (4) IPG: GSES scores reported little change at 4 weeks, and 8 weeks, with scores of 30.8 ± 5.8, and 31.5 ± 6.1 respectively (F = 1.98, *P* = 0.10 > 0.05), and the time impact was not significant.

### Analysis of the impact mechanism of promotion approaches

Regression analysis was utilized to investigate the association between participation in various promotion approaches (e.g., attendance rate, practice frequency), and mental health enhancement (changes in anxiety, depression, and self-efficacy). The analysis of the impact mechanism of participation on mental health enhancement under various promotion approaches is reported in Table 5.

For the correlation analysis in Table 5, a Bonferroni correction was applied to control for the three primary mental health outcomes (anxiety, depression, self-efficacy) tested within each group. The adjusted significance level was set at p < 0.0167 (0.05/3). Unadjusted p-values are shown, with significance after correction denoted as p < 0.0167.

### Correlation between enhancement in anxiety levels (SAS scores), and participation

Regression analysis reported that there was a significant positive association between participation, and enhancement in anxiety levels in Offline-PG, and MPG, while participation in Online-PG, and IPG had a smaller impact on anxiety. Specific results were as follows: (1) Offline-PG: The association between participation, and anxiety enhancement was significant (R = 0.62, *P* = 0.003 < 0.01), determining that participants with higher attendance rates had more significant enhancements in anxiety levels. (2) Online-PG: The correlation between participation, and anxiety enhancement was not significant (R = 0.32, *P* = 0.09 > 0.05), revealing that online independent practice had limited impact on anxiety enhancement. (3) MPG: The association between participation, and anxiety enhancement was significant (R = 0.59, *P* = 0.004 < 0.01), also reporting that high participation had a significant positive impact on anxiety enhancement. (4) IPG: There was no significant correlation between participation, and anxiety enhancement (R = 0.28, *P* = 0.12 > 0.05), determining that independent practice had no obvious impact on anxiety enhancement.

### Correlation between enhancement in depression levels (SDS Scores), and participation

Regression analysis reported that participation in Offline-PG, and MPG was significantly linked to enhancement in depression levels, while the impacts in Online-PG, and IPG were not obvious. Specific results were as follows: (1) Offline-PG: The association between participation, and depression enhancement was significant (R = 0.58, *P* = 0.004 < 0.01), with participants having higher attendance rates reporting significant enhancement in depression levels. (2) Online-PG: There was no significant association between participation, and depression enhancement (R = 0.30, *P* = 0.10 > 0.05), determing that online independent practice had little impact on depressive symptoms. (3) MPG: The association between participation, and depression enhancement was significant (R = 0.55, *P* = 0.005 < 0.01), with participants who had higher participation reporting better impacts in depression enhancement. (4) IPG: There was no significant correlation between participation, and depression enhancement (R = 0.27, *P* = 0.13 > 0.05), revealing that independent practice had no significant impact on depression enhancement.

### Correlation between enhancement in self-efficacy (GSES scores), and participation

In Online-PG, and MPG, there was a significant positive association between the enhancement in self-efficacy, and participation, while participation in Offline-PG, and IPG had less impact on self-efficacy. Specific results were as follows: (1) Offline-PG: The correlation between participation, and enhancement in self-efficacy was not significant (R = 0.29, *P* = 0.08 > 0.05), determining that the On-Site promotion approach had a weak impact on enhancing self-efficacy. (2) Online-PG: Participation was significantly linked to enhancement in self-efficacy (R = 0.67, *P =* 0.002 < 0.01), revealing that participants with higher online participation had more significant enhancements in self-efficacy. (3) MPG: Participation was significantly linked to enhancement in self-efficacy (R = 0.65, *P* = 0.003 < 0.01), determining that the combined online-On-Site participation method had a significant improving impact on self-efficacy. (4) IPG: There was no significant correlation between participation, and enhancement in self-efficacy (R = 0.26, *P* = 0.14 > 0.05), determining that independent practice had little impact on self-efficacy.

## Discussion

Our study determines that various promotion approaches of 24-Style Tai Chi have positively influenced college students’ anxiety, depression, and self-efficacy, but there are significant differences in the effectiveness of each approach On-Site promotion, and online-On-Site mixed promotion models excel in lowering anxiety, and delaying depression. The high attendance rate, and face-to-face interaction environment in Offline-PG may offer more psychological support, thereby significantly lowering participants’ anxiety, and depression [20]. At the same time, the mixed online-On-Site model of MPG offers participants flexible practice options while enhancing effectiveness via On-Site interactions, leading to significant enhancements in anxiety, and depression indicators.

In the context of enhancing self-efficacy, online promotion, and mixed promotion models have obvious advantages. Participants in Online-PG, and MPG can arrange practice time independently, and control their progress flexibly, and such high autonomy helps enhance self-efficacy [21]. This finding suggests that Tai Chi enhanced via flexible online approaches can effectively improve self-efficacy,and integrating On-Site support, and interaction can further enhance intervention impacts. Hence, the potential of 24-Style Tai Chi in college students’ mental health interventions is displayed, and the optimal combination of various promotion approaches can better meet the needs of various students, thereby enhancing the overall intervention effect.

### Analysis of differences in effectiveness of various promotion approaches

Our study reported that various promotion approaches of 24-Style Tai Chi have varying impacts on mental health enhancement, specifically reflected in the differential impacts of On-Site, online, and mixed promotion models on anxiety, depression, and self-efficacy. The following is an in-depth analysis of the advantages, and limitations of each promotion method.

### Benefits of on-site promotion

The on-site promotion model is effective in delaying anxiety, and depression, with its benefits primarily derived from face-to-face interaction, and professional guidance. On-Site courses offer participants with more immersion, and social support, enabling them to receive guidance, and feedback from professional Tai Chi instructors during practice, enhancing emotional support, and a sense of security. This face-to-face guidance enhances participants’ engagement, helping them better perceive the impacts of practice, and relieve psychological stress. In the study, anxiety, and depression levels in Offline-PG significantly reduced, determining that On-Site promotion has obvious benefits in providing emotional support, and psychological intervention [22].

### Limitations of online promotion

Although online promotion offers participants with flexible practice arrangements, it also displays certain limitations, particularly the gradual decline in participation in the later stage of the intervention. Data shows that the participation frequency of Online-PG significantly reduced after 8 weeks, which may lower the maintained impact of the intervention. Because of the lack of face-to-face interaction, and supervision in online promotion, participants’ self-discipline, and practice motivation become key factors impacting effectiveness, causing limited impacts in enhancing anxiety, and depression [23]. Moreover, the online model lacking interactive feedback may make it difficult for participants to obtain immediate guidance, and psychological support, thereby impacting the overall intervention effect.

### Comprehensive benefits of mixed promotion

The mixed promotion model combines the benefits of online, and On-Site approaches, determining high participation while providing flexible practice options, thus reporting significant impacts in enhancing mental health. MPG enables participants to receive professional guidance from instructors, and peer support via On-Site courses, and flexibly arrange practice time utilizing online platforms, enhancing the convenience, and flexibility of participation. This combined online-On-Site approach not only promotes the impacts of anxiety, and depression alleviation, but also significantly enhances participants’ self-efficacy. The dual support mechanism of the mixed promotion model provides participants with sustained practice motivation, and psychological support, making the enhancement impact on mental health particularly significant.

### Impact of participation on intervention effectiveness

Our study reported that participation has a significant impact on the intervention impact on college students’ mental health. Specifically, the higher the participation frequency, the more obvious the enhancement impact on mental health, particularly in terms of anxiety, and depression alleviation. At the same time, the autonomous learning method in online promotion displays unique benefits in enhancing self-efficacy. The following is an analysis of the impact of participation on anxiety, depression alleviation, and self-efficacy enhancement.

### Association between high participation, and significant mental health enhancement

In our study, participation frequency or attendance rate had been central to enhancing anxiety, and depression. The attendance rates of Offline-PG, and MPG were significantly higher than those of other groups, particularly Offline-PG with an attendance rate of 90%, and MPG with 84%. These groups with high attendance rates reported particularly significant enhancements in anxiety, and depression indicators, displaying the significance of high participation in mental health enhancement. On-Site interaction, and instructors’ support enhanced participants’ practice motivation, and effectiveness, enabling them to reach significant emotional regulation, and psychological support via regular participation, thereby significantly lowering anxiety, and depression levels [24]. Conversely, the participation frequency of Online-PG reduced in the later stage, with the attendance rate dropping to 72%, and the participation rate of IPG was even lower, only 60%. These groups with low participation reported insignificant impacts in enhancing anxiety, and depression, determining that lack of sustained participation may weaken the psychological intervention impact of Tai Chi. Hence, high participation is a key factor for significant enhancement in anxiety, and depression; regular participation not only improves the intervention effect, but also helps participants better persist in practice, and consolidate the enhancement of mental health.

### Impact of online participation on self-efficacy enhancement

The results report that autonomous learning in the online promotion model plays a significant role in enhancing self-efficacy, particularly in Online-PG, and MPG with higher participation. Online promotion provides participants with greater time flexibility, enabling them to practice in the context of their own schedules, thereby enhancing autonomy, and a sense of control. This autonomous learning method is tightly linked to the enhancement of self-efficacy, particularly in Online-PG, where the enhancement impact of self-efficacy is significant, determing that participants can better undergo the enhancement of personal abilities, and confidence when arranging practice flexibly [21]. MPG also displays obvious benefits in enhancing self-efficacy; the combined online-On-Site method not only offers the flexibility of autonomous practice, but also takes into account the support of On-Site guidance, further enhancing participants’ self-efficacy. Hence, the autonomy, and flexibility of online participation play a unique role in enhancing self-efficacy, but high participation requires to be sustained to determine the maintained impact of self-efficacy enhancement.

### Comparison with existing studies

The findings of our study are somewhat similar to those of recent literature on common psychological intervention approaches (such as meditation, and exercise therapy) in enhancing mental health, but 24-Style Tai Chi displays its uniqueness, and benefits in specific intervention mechanisms, and enhancement impacts.

### Comparison with meditation, and exercise therapy

In the realm of psychological intervention, meditation, and exercise therapy have been commonly utilized to promote anxiety, depression, and self-efficacy. Meditation helps individuals relax via concentration, and breathing regulation, thereby enhancing emotional regulation ability; exercise therapy releases pressure via physical activity, and enhances participants’ sense of well-being, and self-identity. These intervention approaches are generally effective in the short-term alleviation of anxiety, and depression, but have limited impacts in enhancing self-efficacy [25].

It is worth noting that compared with meditation, and exercise therapy, the Tai Chi intervention in our study not only performs well in delaying anxiety, and depression, but also displays significant impacts in enhancing self-efficacy. The slow, and coherent movements of Tai Chi, stressing the coordination of body, and breathing, help participants find inner peace in stability, obtaining the impacts of relaxation, and emotional regulation [26,27]. Moreover, the sense of physical control enhanced via Tai Chi movements further improves participants’ self-efficacy [28]. This inner confidence obtained via physical control is in sharp contrast to additional psychological intervention approaches, making Tai Chi have stronger benefits in long-term mental health enhancement. Moreover, the online, and On-Site promotion approaches of Tai Chi offer participants with flexible participation opportunities, enabling them to better integrate into daily life. Conversely, meditation, and exercise therapy generally need independent venues, and time arrangements, while Tai Chi has stronger adaptability in promotion. Moreover, the social, and interactive nature of Tai Chi (particularly in On-Site promotion) offers participants with additional psychological support, hence enhancing the intervention effect.

### Limitations of our study

The sample size of our study is relatively limited. Although we recruited enough participants in each group to determine statistical power, a larger sample size may offer more accurate, and robust results. Moreover, the experimental period of our study is 8 weeks, and the short intervention time may not completely imply the long-term impacts of Tai Chi on mental health. Future studies can expand the experimental period to uncover the maintained impact of Tai Chi on mental health over a longer period. Our study primarily counts on participants’ self-reports to analyze mental health indicators, such as anxiety, depression, and self-efficacy, which may introduce subjective bias. Participants’ emotional states, expectation impacts, or social desirability bias may impact the accuracy of reports, particularly in mental health scores change the intervention. Hence, future studies can consider integrating objective physiological indicators, such as heart rate variability, and cortisol levels, to promote the objectivity of mental health evaluation. Furthermore, it is noteworthy that our study was only conducted on college students, in detail, although college students are a group with high mental health needs, this sample may not represent other age groups or occupational groups. Hence, the extrapolation of research results to other populations is limited. Individuals with various ages, and occupational backgrounds may have significant differences in participation, practice preferences, and psychological responses. Future studies can investigate whether the results apply to various populations to improve the universality, and promotion of results. Fourth, our study experienced varying attrition rates across groups, which is an important limitation. The high attrition in the IPG (40%) and Online-PG (28%) groups, compared to the Offline-PG (10%) and MPG (16%) groups, likely reflects the inherent challenge of sustaining self-motivated practice without structured supervision or social interaction. This differential attrition underscores the critical importance of guidance and social support in maintaining adherence to mind-body exercise programs. While our regression analyses accounted for participation levels, the substantial dropout in less-structured groups may have introduced bias and limited our ability to detect significant effects in those conditions. Future studies should implement strategies such as reminder systems, motivational interviewing, or minimal social support to enhance retention in independent or online practice models.

### Practical significance, and suggestions

Our study displays that 24-Style Tai Chi has good application prospects in college mental health interventions. As a conventional sport integrating physical activity, and psychological regulation, Tai Chi not only helps delay anxiety, and depression, but also enhances students’ self-efficacy. Promoting Tai Chi as a mental health intervention in colleges, and universities can not only act as a strong supplement to conventional psychological services, but also enrich students’ extracurricular activities, and cultivate their awareness of physical, and mental health. To further enhance the promotion effect, the following feasible suggestions for promotion approaches are put forward: (1) Enhance the interest of courses, such as introducing graded practice, dynamic challenges, or achievement systems to improve participants’ sustained interest, and engagement; (2) Online promotion can add social interaction or feedback functions, such as virtual instructors, peer encouragement, and real-time feedback, to help participants better track progress, and obtain motivation; (3) To improve participation, consider designing diversified promotion forms, such as theme courses, short-term challenge activities, or online-On-Site mixed promotion, to enhance the flexibility, and attractiveness of practice. The above optimization suggestions aim to improve students’ acceptance, and sustained participation in Tai Chi, determining the sustained impact of mental health enhancement [6].

### Future research directions

Our study provides empirical support for the impact of 24-Style Tai Chi in enhancing college students’ mental health, but future studies can further investigate its impacts in various populations, and intervention approaches. (1) Future studies can verify the mental health impacts of Tai Chi in other populations (such as office workers, middle-aged, and elderly people, and adolescents) to investigate the adaptability, and impact differences in various populations; (2) The intervention period of the current study is 8 weeks; future studies can extend the intervention time to analyze the impact of Tai Chi in long-term mental health enhancement, and observe its sustained role in mental health; (3) It is suggested to further study the potential promoting impact of online interaction, and feedback mechanisms on intervention impacts, and investigate how factors, such as virtual instructors, real-time feedback, and social support affect the enhancement of mental health in online promotion.

## Conclusion

Our study displays that various promotion approaches have significantly various impacts on enhancing college students’ mental health. The participation rates of On-Site promotion, and mixed promotion are higher. On-Site promotion, and mixed promotion have significant impacts on enhancing anxiety, and depression levels, and sustain good impacts during the follow-up stage; online promotion, and mixed promotion have significant impacts on enhancing self-efficacy, while independent practice has the weakest enhancement effect. There are significant inter-group differences in the impacts of various promotion approaches on mental health enhancement; On-Site promotion, and mixed promotion are particularly prominent in enhancing anxiety, and depression, while online promotion, and mixed promotion have significant benefits in enhancing self-efficacy; the impact of independent practice is not significant in all indicators. The anxiety, and depression levels of On-Site promotion, and mixed promotion decrease significantly over time, with the best impact at 8 weeks of intervention; the self-efficacy of online promotion, and mixed promotion increases significantly with the intervention time, while independent practice has no significant time effect. There is a significant positive association between the participation of On-Site promotion, and mixed promotion, and the enhancement of anxiety, and depression; high participation significantly enhances the enhancement impact of anxiety, and depression; the participation of online promotion, and mixed promotion is significantly linked to the enhancement of self-efficacy, determining that online interaction, and mixed participation modes have obvious impacts on enhancing self-efficacy; the participation of independent practice has no significant impact on mental health.

## Supporting information

S1 File Statistical analysis results. (DOCX)

S1 Dataset Dataset used for analyses in present study. (XLSX)

S1 Checklist CONSORT_2025_editable_checklist. (DOCX)
