# Supplementary material for: Comparison of the 24-Style Tai Chi intervention based on various promotion approaches on college students’ mental health: A randomized controlled trial
Source: PLoS One. 2026 Feb 27;21(2):e0343808. doi: 10.1371/journal.pone.0343808 (PMC12948128; doi:10.1371/journal.pone.0343808)
Supplement: S1 File — (DOCX) [file pone.0343808.s001.docx]

**Table 1. Comparison of 24-Style Tai Chi Participation, and Baseline Mental Health features Under various Promotion approaches**

| **Group** | **Offline-PG** | **Online-PG** | **MPG** | **IPG** | **CG** |
| --- | --- | --- | --- | --- | --- |
| Average Attendance/Participation Rate | 90% | 72% | 84% | 60% | N/A |
| Original/Final Number of Participants | 50/45 | 50/36 | 50/42 | 50/30 | 50/50 |
| Participation in Follow-up Phase | Most participants maintained  participation at least twice a week | Participation frequency decreased over time | Maintained a high level of participation | Some participants dropped out midway | No intervention |
| SAS Baseline Score (M ± SD) | 44.1 ± 8.0 | 44.5 ± 8.3 | 44.4 ± 8.1 | 44.2 ± 8.5 | 44.3 ± 8.4 |
| SDS Baseline Score (M ± SD) | 46.3 ± 9.3 | 46.0 ± 9.6 | 46.4 ± 9.4 | 46.1 ± 9.7 | 46.2 ± 9.5 |
| GSES Baseline Score (M ± SD) | 29.6 ± 5.7 | 29.8 ± 5.9 | 29.9 ± 5.6 | 29.5 ± 5.8 | 29.7 ± 5.8 |
| Inter-group t-test P-value (SAS) | ＞ 0.05 | ＞ 0.05 | ＞ 0.05 | ＞ 0.05 | ＞ 0.05 |
| Inter-group t-test P-value (SDS) | ＞ 0.05 | ＞ 0.05 | ＞ 0.05 | ＞ 0.05 | ＞ 0.05 |
| Inter-group t-test P-value (GSES) | ＞ 0.05 | ＞ 0.05 | ＞ 0.05 | ＞ 0.05 | ＞ 0.05 |

**Table 2**. **Impact of 24-Style Tai Chi on Changes in College Students’ Mental Health Indicators Under various Promotion approaches**

| **Mental Health Indicators** | **Offline-PG(n = 45)** | **Online-PG(n = 36)** | **MPG(n = 42)** | **IPG(n = 30)** | **CG(n = 50)** |
| --- | --- | --- | --- | --- | --- |
| SAS Score (4 weeks) | 38.9 ± 6.8 | 41.3 ± 7.6 | 39.7 ± 7.2 | 41.7 ± 7.9 | 44.3 ± 8.4 |
| SAS Score (8 weeks) | 34.8 ± 6.3 | 39.5 ± 7.8 | 36.2 ± 7.0 | 40.1 ± 8.0 | 44.3 ± 8.4 |
| SAS Score (Follow-up) | 35.1 ± 6.5 | 40.2 ± 7.9 | 36.5 ± 7.1 | 40.3 ± 8.2 | 44.3 ± 8.4 |
| SDS Score (4 weeks) | 37.4 ± 7.2 | 40.5 ± 7.8 | 38.2 ± 7.5 | 40.9 ± 7.9 | 46.2 ± 9.5 |
| SDS Score (8 weeks) | 33.7 ± 6.9 | 38.6 ± 7.5 | 34.9 ± 7.2 | 39.3 ± 7.9 | 46.2 ± 9.5 |
| SDS Score (Follow-up) | 34.0 ± 7.1 | 39.0 ± 7.6 | 35.1 ± 7.3 | 39.4 ± 8.0 | 46.2 ± 9.5 |
| GSES Score (4 weeks) | 31.7 ± 5.8 | 33.9 ± 5.5 | 33.7 ± 5.4 | 30.8 ± 5.8 | 29.7 ± 5.8 |
| GSES Score (8 weeks) | 32.8 ± 5.9 | 36.1 ± 5.2 | 35.5 ± 5.6 | 31.5 ± 6.1 | 29.7 ± 5.8 |
| GSES Score (Follow-up) | 32.7 ± 5.8 | 35.8 ± 5.3 | 35.3 ± 5.5 | 31.6 ± 6.0 | 29.7 ± 5.8 |

**Table 3**. **Analysis of Differences in Mental Health Indicators in the context of**

| **Comparison** | **Anxiety (SAS Score)** | **Depression (SDS Score)** | **Self-Efficacy (GSES Score)** |
| --- | --- | --- | --- |
| One-Way ANOVA (Overall) | F = 6.45, p = .004 | F = 5.32, p = .009 | F = 6.74, p = .003 |
| Post-hoc p-values  vs. Control Group (CG) |  | | |
| Offline-PG vs. CG | .004 | .009 | .090 |
| Online-PG vs. CG | .120 | .110 | .003 |
| MPG vs. CG | .007 | .013 | .005 |
| IPG vs. CG | .150 | .160 | .130 |
| Between Intervention Groups |  | | |
| Offline-PG vs. Online-PG | .018 | .025 | .010 |
| Offline-PG vs. MPG | .210 | .185 | .022 |
| Offline-PG vs. IPG | .002 | .001 | .135 |
| Online-PG vs. MPG | .045 | .055 | .285 |
| Online-PG vs. IPG | .320 | .275 | .001 |
| MPG vs. IPG | .009 | .007 | .005 |

**Groups Under various Promotion approaches**

Footnote for Table 3:

Abbreviations: PG, Promotion Group; MPG, Mixed Promotion Group; IPG, Independent Practice Group; CG, Control Group.

The one-way ANOVA was used to test the overall effect across all five groups. Post-hoc pairwise comparisons were conducted only if the overall ANOVA was significant. To account for multiple comparisons, a Bonferroni correction was applied, setting the family-wise significance level at p < 0.05. The specific number of comparisons and adjusted alpha levels were as follows: for the four pre-planned comparisons of each intervention group vs. the control group, the adjusted significance level was p < 0.0125 (0.05/4); for all possible pairwise comparisons between groups, the adjusted level was p < 0.005 (0.05/10). Unadjusted p-values are presented in the table, with asterisks denoting significance after Bonferroni correction: p < 0.0125, p < 0.005.

**Table 4. Time impact Analysis of Mental Health Indicators in Each Group Under various Promotion approaches**

| **Group** | **Anxiety (SAS)** | **Depression (SDS)** | **Self-Efficacy (GSES)** |
| --- | --- | --- | --- |
| Time Effect (RM-ANOVA) |  |  |  |
| Offline-PG | F = 8.22, p = .003 | F = 7.93, p = .002 | F = 2.05, p = .090 |
| Online-PG | F = 2.11, p = .090 | F = 2.23, p = .080 | F = 7.58, p = .004 |
| MPG | F = 7.45, p = .004 | F = 6.89, p = .005 | F = 6.45, p = .006 |
| IPG | F = 1.98, p = .110 | F = 1.77, p = .120 | F = 1.98, p = .100 |

Footnote for Table 4:

RM-ANOVA = Repeated Measures Analysis of Variance.

For the correlation analysis in Table 5, a Bonferroni correction was applied to control for the three primary mental health outcomes (anxiety, depression, self-efficacy) tested within each group. The adjusted significance level was set at p < 0.0167 (0.05/3). Unadjusted p-values are shown, with significance after correction denoted as p < 0.0167.

**Table 5. Analysis of the impact Mechanism of Participation on Mental Health enhancement Under various Promotion approaches**

| **Group** | **Anxiety (SAS)** | **Depression (SDS)** | **Self-Efficacy (GSES)** | **Group** |
| --- | --- | --- | --- | --- |
| Offline-PG | R = 0.62, p = .003 | R = 0.58, p = .004 | R = 0.29, p = .080 | Offline-PG |
| Online-PG | R = 0.32, p = .090 | R = 0.30, p = .100 | R = 0.67, p = .002 | Online-PG |
| MPG | R = 0.59, p = .004 | R = 0.55, p = .005 | R = 0.65, p = .003 | MPG |

Footnote for Table 5:

RM-ANOVA = Repeated Measures Analysis of Variance.

For the correlation analysis in Table 5, a Bonferroni correction was applied to control for the three primary mental health outcomes (anxiety, depression, self-efficacy) tested within each group. The adjusted significance level was set at p < 0.0167 (0.05/3). Unadjusted p-values are shown, with significance after correction denoted as p < 0.0167.
